# The stress concept in gastroenterology: from Selye to today

**DOI:** 10.12688/f1000research.12435.1

**Published:** 2017-12-19

**Authors:** Sigrid Elsenbruch, Paul Enck

**Affiliations:** 1 Institute of Medical Psychology & Behavioral Immunobiology, University Hospital Essen, University of Duisburg-Essen, Essen, Germany; 2 Department of Internal Medicine VI: Psychosomatic Medicine and Psychotherapy, University Hospital Tübingen, Tübingen, Germany

**Keywords:** gastroenterology, stress, gut microbiota

## Abstract

More than eighty years after Hans Selye (1907–1982) first developed a concept describing how different types of environmental stressors affect physiological functions and promote disease development (called the “general adaptation syndrome”) in 1936, we herein review advances in theoretical, mechanistic, and clinical knowledge in stress research, especially in the area of gastroenterology, and summarize progress and future perspectives arising from an interdisciplinary psychoneurobiological framework in which genetics, epigenetics, and other advanced ( *omics*) technologies in the last decade continue to refine knowledge about how stress affects the brain-gut axis in health and gastrointestinal disease. We demonstrate that neurobiological stress research continues to be a driving force for scientific progress in gastroenterology and related clinical areas, inspiring translational research from animal models to clinical applications, while highlighting some areas that remain incompletely understood, such as the roles of sex/gender and gut microbiota in health and disease. Future directions of research should include not only the genetics of the stress response and resilience but also epigenetic contributions.

## Introduction

Our review will start with a short historical vignette on Hans Selye’s contribution to our current understanding of the concept of environmental stressors on human disease and will bridge to acute research questions driven by progress in neurophysiology (“decade of the brain”) and, more recently, microbiology. In three sections, we will then elaborate how stress research has contributed to basic animal studies in gastroenterology (for example, on the role of sex differences and the contribution of the gut microbiota for understanding the stress response and visceral hypersensitivity, in translational research on the commonalities and differences between acute and chronic stress in humans, and on clinical research exploring whether and how stress contributes to functional and other gastrointestinal [GI] disorders, taking both basic [sex and microbiota] and technical [brain imaging] aspects into consideration).

## Historical vignettes

In the July issue of the journal *Nature* in 1936, 29-year-old Hans Selye, a Vienna-born Austrian-Hungarian who studied medicine and chemistry in Prague, Paris, and Rome before completing his Ph.D. at Johns Hopkins University and immigrating to Montreal, published his first (!) paper. This short note entitled “A syndrome produced by diverse nocuous agents” ^1^ was about twice the size of an abstract nowadays, yet the syndrome would later become known as the stress concept, also known as “general adaptation syndrome” (GAS). Although it described the major principle, a global and homogenous three-phase bodily response to a variety of different noxious stimuli, the term “stress” was not mentioned. It also contained no reference that this concept may be of any special relevance to the GI tract, except that Selye noted that “the formation of acute erosions in the digestive tract, particular in the stomach, small intestine and appendix” of the animals (rats) following exposure to noxious agents occurred ^1^. Ten years later, Selye published a full account of his experimental findings, entitled “The general adaptation syndrome and diseases of adaptation” ^2^, that may mark the true beginning of the GAS/stress theory, again remarkable for different reasons: for the fact that this paper was published simultaneously in several journals ( *Journal of Allergy, Annales d’Endocrinologie*, *Manpower*, *Piersol’s Cyclopedia of Medicine, Surgery and Specialties*, and *Bulletin de Biologie et de Médecine Expérimental de l’U.R.S.S.*), which is entirely impossible to think of nowadays, and for the frequently reproduced figure illustrating the—at that time—unknown pathways connecting the brain to peripheral bodily systems, including the GI tract. Yet it was GI physiology and the search for pathways and their neuroendocrine mediators, including those involved in “stress ulcers” in the gut, that subsequently received the most attention: UCLA’s Center for Ulcer Research and Education ^3^, founded in 1974, was the Mecca for stress research outside its hub in Montreal, Canada. This promoted the idea that central stress causes or contributes to many peripheral diseases—a concept that ever since has been discussed in gastroenterology, much earlier than in other core medical areas and subspecialties. Ulcers are no longer a major focus of stress research in gastroenterology, but, given the detection of *Helicobacter pylori* and its involvement in ulcer formation, stress research in gastroenterology continues to thrive.

Seventy years after Selye’s account and at the end of the “Decade of the Brain”, the September 2015 issue of *Nature Neuroscience* provided state-of-the-art reviews of stress research summarizing the remarkable progress in our understanding of mechanisms involved in central processes and their clinical implications for multiple diseases and health conditions, ranging from psychiatric to cardiovascular and immune-related diseases. Important conceptual developments, especially the concepts of allostasis and allostatic load ^4^, continue to provide a more refined psychoneurobiological framework to explain the mechanisms and clinical implications of chronic stress and stress-related conditions. These incorporate new aspects such as the role of threat perception, cognitions, coping, and appraisal processes ^5, 6^ with a focus on mental health, individual variability, and resilience ^7, 8^ and their underlying neurobiological mechanisms.

Today, stress research is highly transdisciplinary and has many facets, including research into motivation and reward, plasticity, cognition, and sex differences, to name a few. Some of these topics have found their way into gastroenterological research; others have yet to be incorporated. Although recent work is carried out mostly in the context of visceral pain ^9^ and the biopsychosocial disease model in functional GI disorders such as irritable bowel syndrome (IBS) and functional dyspepsia (FD), interest in stress and biopsychosocial disease concepts ^10, 11^ has started to extend to other GI conditions such as inflammatory bowel diseases (IBDs) ^12, 13^, liver diseases ^14^, and celiac disease ^15, 16^.

In the following, we will discuss current facets of stress research both in animal studies and in human research and will outline its relevance for the pathophysiology of GI conditions, either shown or proposed.

## Translational approaches to study acute and chronic stress

To reliably produce gastric (stress) ulcers, a simple cold-restraint model was used until the 1980s in most animal studies, for example, ^17^, but was frequently questioned for its relevance in humans and replaced by other stressors (for example, by noise ^18^) when GI functions (motility and secretion) rather than ulcer formation were of interest. But it was not until in 1989, when a truly psychological (that is, non-invasive and non-physical) stress model for rodents—the water avoidance model ^19^—was introduced, that animal stress research became truly relevant for the investigation of intestinal functions and dysfunctions in humans. Yet other animal models—neonatal maternal separation ^20^ and, more recently, limited nesting ^21^—sparked the initiation of a large series of studies on the long-term effects of stress on visceral sensitivity and related dysfunctions in animals.

In humans, there are a number of approaches to study the effects of acute and chronic stress and underlying psychological and neurobiological mechanisms. One prominent example of a well-established acute laboratory stress model is the Trier Social Stress Test (TSST), which combines a difficult cognitive task (mental arithmetic) with a public-speaking task in front of an audience. The TSST is a widely established, highly standardized, and purely psychosocial trigger of acute stress responses ^22, 23^ that reliably induces pronounced yet transient increases in psychological and biological stress markers, including emotional and cognitive responses along with activation of the hypothalamus-pituitary axis and sympathetic nervous system. Several examples for its application in the context of the GI system exist ^24– 30^, while other work in the field ^31– 34^ has implemented alternative approaches to induce psychological stress. Some of these experimental protocols produce weaker, less reliable stress effects (for example, dichotomous listening), incorporate a physical pain component (for example, cold pressure test), or focus primarily on emotional or cognitive aspects (for example, listening to sad music, seeing disturbing pictures, and anticipating electric shock). Pharmacological approaches, such as the administration of corticotropin-releasing hormone (CRH), CRH antagonist, or hydrocortisone, which have recently been accomplished in the GI system ^35– 38^, allow clinicians to specifically assess effects on GI-related functions mediated by the hypothalamic-pituitary-adrenal (HPA) axis but arguably have limited external validity as models of psychological stress in humans given a lack of effects at the subjective level (for example, no increase in subjective stress levels of state anxiety).

In contrast to acute stress, which induces an adaptive response preparing the organism for “fight-or-flight” and therefore is not harmful per se, chronic stress evokes maladaptive psychophysiological changes which, when severe, can have a multitude of clinical ^39^ and broad implications ^40, 41^ for the GI system. It is defined as the psychophysiological response to long-term emotional pressures such as adverse life events over which the individual perceives little or no control and typically is measured with validated questionnaires (for example, the Trier Inventory for the Assessment of Chronic Stress [TICS] ^42^ and the Perceived Stress Questionnaire ^43^).

Although experimental approaches in animals and humans are divergent and continue to evolve, broad knowledge about centrally mediated effects of stress on GI sensorimotor functions has fundamentally shaped the concept of the brain-gut axis and continues to inspire animal and human studies.

## Current animal stress research in the gastrointestinal tract

Visceral hypersensitivity—an abnormally high responsiveness of the gut toward physiological stimuli (for example, distension)—is regarded as a key feature of functional bowel disorders of IBS type ^44^. In animals, it can reliably be induced by a temporary (for example, early life) exposure of a gut segment to a noxious but transient stimulus that leaves the segment unaltered morphologically but responsive to low-level stimuli later in life ^45^ and other, non-GI stimuli (for example, foot-shock) work as well ^46^. Visceral hypersensitivity can also be induced in newborn pups when they are exposed to maternal separation (1 hour per day for a week or two) and are retested days, weeks, or months later ^20^; this effect appears specific for visceral hypersensitivity but not for other behavioral measures ^47^. Such an effect of early life stress is not limited to rodents but also occurs in other mammals, such as in porcine models where it induced chronic functional diarrhea and intestinal barrier defects and increased mast cell activity ^48^, lasting hypersensitivity of secretomotor neuron function, and upregulation of the cholinergic enteric nervous system ^49^.

Neonatal maternal separation also changes neurocognitive functions ^50^ and stress responsiveness in the dams ^51^; whether visceral sensitivity of the mothers is altered remains unknown. When pregnant rats are exposed to a gut-sensitizing stimulus, their offspring will also show visceral hypersensitivity ^52^. It has been shown that such experimentally induced hypersensitivity will be transmitted across generations ^53^, indicating “soft” rather than Mendelian inheritance and an epigenetic mechanism for this ^54^. Whether transmission of susceptibility occurs via transmission of hormonal concentrations to offspring via lactation ^55^ or via alterations of the gut microbiota that is transmitted vertically ^56^ remains an open issue.

Even if gut segments of stress-exposed animals show little or no morphological alterations upon macroscopic or microscopic inspection, they still may behave differently not only *in vivo* but also *ex vivo* when jejunal and colonic segments of animals stressed by restraint for one hour demonstrated decreased motility frequency and increased amplitude *in vitro* ^57^. According to the authors, this implies that dysmotility is generated by mechanisms internal to the gut (rather than central), presumably via immune-mediated or neurally mediated changes of the enteric nervous system, because of the short-term nature of the stress-test interval. One putative mediator may be neuropeptide Y (NPY); its receptors play important roles in—among others—stress resilience ^58^.

The variability of stress responses in different animal strains of the same species—for example, selective breeding-based cholinergic hypersensitivity and hyposensitivity Flinders rat lines ^59^ or hyperanxious (HAB-M) and hypoanxious (LAB-M) mouse lines ^60^—or increased stress responsiveness in Wistar Kyoto rats, as compared with Sprague Dawley rats ^61^, is well established. The importance of individual vulnerability and resilience factors is increasingly acknowledged both conceptually (for example, ^8^) and in mechanistic research and may exhibit a genetic ^62^ and an epigenetic ^63^ basis, and this is possibly based on “synaptic rewiring” of stress-sensitive neurons ^64^. In all cases, however, it is likely that the “three-hit concept” of vulnerability and resilience persists: a genetic predisposition and early life adverse events are necessary so that a later-in-life stressor can exhibit negative health outcomes, and one or more missing may result in higher resilience ^65^. It is of importance to note that resilience has not yet been thoroughly investigated in relation to GI functions in animals (and humans) under stress; it is, however, known that patients with IBS lack resilience, and low resilience was associated with worse IBS severity, lower quality of life, more early life stressful events, and stress hyper-responsiveness ^66^. Similarly, in patients with IBD, the role of (maladaptive) coping is only beginning to be unraveled (for example, ^67– 69^), calling for translational research on individual risk and resilience in patients with GI conditions.

## Sex differences in rodents and humans

Gender differences in the prevalence of chronic visceral pain, especially a female preponderance of functional gastrointestinal disorders (FGIDs), are well established. Further support for a role of sex-related factors comes from mechanistic human and animal research showing sex differences in visceral pain processing in animal models, healthy individuals, and patients with FGIDs ^70, 71^. The putative connection linking gender/sex and sex hormones to stress and pain is undoubtedly highly complex yet intriguing and in need of more dedicated research in animal models, healthy humans, and patients ^70, 72^ with attention to effects across the life span ^73^. After all, many sex differences exist in the central and peripheral response to stress because of dimorphic brain development ^73^. During gestation, sex differences in embryonic responses to maternal and environmental stress are well documented, and males are at higher risk for negative outcomes. In humans, this is associated with higher incidences of neurological disorders (attention-deficit/hyperactivity disorder, among others); in animals, stress during pregnancy predominantly affects male offspring ^74^. During childhood, in contrast, stress appears to increase the risk for affective disorders, and here women are at higher risk, especially during their reproductive years. Whether this explains the higher incidence of functional (GI) disorders remains an open issue, as this is dependent also on the effects of prenatal and perinatal stress on the development of intestinal functions that have rarely been investigated in this context.

Preliminary data suggested a strong sex difference in some of the reported consequences of stress on intestinal functions, and females were more resilient in general than males. Both chronic and intermittent stress models (for example, limited nesting) have profound consequences on the offspring with minimal external intervention from the investigator ^75^. Limited nesting of rat dams increased gut permeability predominantly in female Wistar pups, but overall stress-decreased diversity of the gut microbiota was similar between sexes ^56^; in another study from the same group, offspring male pups showed increased gut permeability but female pups did not ^76^. Water-avoidance stress reduced the visceral motor response to colorectal distension immediately after the stressor, and this analgesic effect was opioid-dependent (naloxone-sensitive) in females but insensitive to naloxone in males, and repeated stress induced hyperalgesia in females only ^77^. Sexual dimorphism was also found in mast cell responses to stress, with female mice “exhibiting increased clinical scores, hypothermia, and serum histamine levels in response to stress and greater intestinal permeability and serum histamine responses” ^78^. In the above-cited porcine model ^48, 49^, responses in females overall were larger than in male animals.

## The role of stress in patients

Patients with FGIDs report higher levels of chronic stress and more adverse life events, and the proportion of patients who present with a history of early life stress or trauma is considerable ^79, 80^. In prospective studies, chronic stress has been identified as one of the psychological risk factors for the development of an FGID later in life or for post-infectious IBS; in IBD, chronic stress prospectively increases the risk of relapse ^12^, but the connection between GI symptom (reports), intestinal inflammation, and stress remains to be clarified ^81^. Importantly, stress and other psychological disturbances such as depression or anxiety symptoms can both precede the manifestation of chronic GI complaints and occur as a consequence of the GI condition ^82^, supporting a complex interplay between psychological changes and GI symptoms in terms of a vicious cycle.

The ability of acute stress, acute negative emotions, or HPA-axis mediators to influence both upper and lower GI sensorimotor processes and central pain processing has been extensively documented in healthy humans ^79^. In patients with FGID, knowledge is not as extensive, but stress effects appear to be altered, especially in patients with hypersensitivity. For example, in patients with FD, state anxiety at the time of testing was associated with impaired gastric accommodation ^83^ and correlated negatively with gastric discomfort and pain thresholds and with gastric compliance in hypersensitive FD ^84^. Mental stress failed to produce the normal reduction in antral motility in patients with FD ^30, 85^. The neurobiological mechanisms underlying these effects remain incompletely understood, especially in patients, but likely involve both brain mechanisms and top-down neuroendocrine and autonomic pathways and may include mast cell-dependent effects on permeability ^30, 79, 86^.

## Brain mechanisms

Brain imaging studies have started to delineate the neural mechanisms underlying the effects of stress and other psychological variables on visceral sensation and central pain processing ^79, 87– 89^. For example, acute stress or negative mood demonstrably alters distension-induced neural activation in multiple brain regions, including the insula, cingulate cortex, and prefrontal areas, in healthy individuals and patients with IBS ^90, 91^. In FD, anxiety during scanning reportedly contributes to group differences between patients and healthy controls ^92^. In IBS, effects of acute stress on central pain processing were more pronounced in specific brain regions ^25^. Changes in central nervous pain processing in IBS have further been shown to be associated with anxiety symptoms and depression ^91^, symptoms which are distinct from chronic stress but illustrate the broad role of both chronic and acute psychological factors. Interestingly, patients with IBS also exhibit altered brain activation during pain anticipation ^89^. Such anticipatory responses—mainly in brain areas linked to attention, threat detection, and emotion regulation—reflect pain-related fear resulting from associative learning processes ^37, 93^, which influence the processing of visceral stimuli even in healthy humans ^94^. In patients with IBD, brain imaging studies have only recently begun to emerge ^95, 96^, including studies addressing effects of acute stress ^97, 98^, laying the foundation for much-needed research on putative similarities and differences in structure-function relationships along the brain-gut axis in IBD and IBS.

## Pain-related learning and memory processes

Stress may contribute to impaired pain-related learning and extinction processes and thereby play a role in the transition from acute to chronic pain or the maintenance of chronic symptoms or both. The conceptual basis for this assumption is evidence that functional and structural brain alterations involved in the pathophysiology of chronic pain overlap with brain circuits involved in emotion regulation and stress ^99^ and with regions mediating fear expression and recovery ^100^. From a learning perspective, recurrent painful episodes induce associative and instrumental learning processes. The putative clinical relevance is supported by evidence that learning-based treatment approaches, particularly of exposure-based interventions, are efficacious in IBS ^101, 102^ and other chronic pain conditions ^103^. Based on mechanistic work, it has been proposed that conditioning may lower pain thresholds ^104^ or promote sensitization ^105, 106^ and thus contribute to hyperalgesia or hypervigilance or both, impair perceptual discrimination acuity ^107^, enhance fear generalization ^108^, or interfere with normal habituation processes ^109^, but some of these suggestions come from studies implementing somatic rather than visceral stimuli. To unravel the mechanisms engaged in pain-related associative learning, new research studies have implemented innovative experimental paradigms with visceral stimuli such as unconditioned stimuli or conditioned stimuli (or both) in healthy individuals and patients with IBS ^93^, some of them using brain imaging techniques to address underlying neural mechanisms ^37, 110^. However, virtually nothing is known about the possible roles of affective comorbidity and stress in shaping disturbed acquisition or impaired extinction of pain-related fear. Applying existing findings regarding the effects of stress or HPA-axis mediators such as cortisol on memory consolidation and reconsolidation to the field of GI, one could postulate that stress results in a reactivation of the pain-related memory trace or facilitates its reconsolidation or both, ultimately making the pain-related fear memory more lasting. This process may contribute to the maintenance of pain-related fear and hypervigilance and thereby to maladaptive avoidance behavior as part of a vicious circle maintained by stress and fear ^111^. Furthermore, research into interactions between affective comorbidity, acute stress, and memory processes may contribute to elucidating individual risk and vulnerability factors and neuropharmacological treatment options for chronic pain ^112^. In addition to the many options available to modify stress responses at the central level via medical and psychological strategies, nutritional interventions have recently found increased attention.

## Stress and microbiota

Stress induces alterations of the fecal microbiota, and manipulation of the gut microbiota alters stress responses, in both humans and animals. Experimental stress in animals showed sustained alterations of the gut microbiome across species ^113^. Stress in pregnant mice disrupted that natural patterning of the gut microbiota during pregnancy. The disruption was observed not only in the gut microbiota but also in the vaginal microbiota ^114^; gut microbiota disruption may influence maternal nutritional status and thus change the energy supplies available to the brain of the developing offspring. The development of sexual dimorphism, discussed above, is presumably driven by sex differences in the gut microbiome–brain axis across the life span ^115^.

In humans, stress-associated disorders have been characterized by altered microbiota profiles—for example, in post-traumatic stress disorder ^116^, IBS ^117^, depression ^118^, eating disorders such as anorexia nervosa ^119^, and other psychiatric or neurological central nervous system (CNS)-related disorders ^120^. Acute exercise affects the microbiota via mitochondrial mediation ^121^, and long-term stress exposure altered intestinal permeability and microbial composition ^122^. Professional athletes show moderately altered microbiota profiles but significantly increased metabolic activity (short-chain fatty acids, acetate, and butyrate) compared with sedentary adults ^123^, and similar differences were found between an active and a sedentary lifestyle in women ^124^. A correlation between cardiovascular fitness and microbiota composition was also found in breast cancer survivors ^125^.

We have recently reviewed the literature on probiotic effects in CNS functions in animals and humans ^126^ and found rather inconsistent results. The effects depended on, among other things, the bacterial species applied and the CNS function under investigation, and some positive effects in animals with a specific strain ^127^ were not replicable in humans with the same strain ^128^. When the probiotic *Lactobacillus rhamnosus* JB-1 was applied locally in *ex vivo* gut segments, it reversed restraint stress-induced gut dysmotility ^57^. In addition, similar strains may exhibit different responses; for example, the *Lactobacillus pentosus* strain S-PT84 showed anti-stress activity and ameliorated stress-induced immune suppression in mice ^129^, while another *Lactobacillus* strain, *Lactobacillus casei* 54-2-33, might have anxiogenic effects in mice ^130^. Yet another *Lactobacillus* strain reversed stress-induced cognitive, behavioral, and biochemical alterations in rats ^131^, but a similar effect was seen with strain-unspecific dietary interventions (for example, with polyunsaturated fatty acids) ^132^. In chronically stressed mice, restoring stress-decreased *Lactobacillus* abundance in the gut microbiota reversed behavioral alterations ^133^, and oral intake of *Bifidobacteria* significantly increased the number of resilient mice compared with vehicle-treated mice in another stress model ^134^. Also, prebiotic pretreatment of animals prolonged stress-induced visceral analgesia following colorectal distension ^135^, and this was associated with a reduction of cecal content of isobutyrate and total butyrate. It had anxiolytic effects and reversed the impact of chronic stress in mice ^136^. However, it should be kept in mind that these experiments were frequently performed in germ-free animals colonized by single bacterial species, or complex microbiota transplanted from other animals, or “humanized” with fecal microbiota from healthy or diseased humans. Germ-free mice by themselves are questionable models for regular human gut ecology, and elimination or distortion of the gut microbiota by antibiotics is feasible only in animals, except with the locally acting antibiotic rifaximin that exerted stress-reducing effects in healthy volunteers ^137^ in a stress paradigm mimicking social isolation ^138^.

Some *Bifidobacteria* exert strain-specific beneficial effects on stress-related behavior ^139^ and cognitive functions in mice ^140^ and in healthy humans ^141^ and may be potential candidates for the management of patients with IBS ^142^. In healthy humans, the *L. casei* strain Shirota preserved the diversity of the gut microbiota and relieved abdominal dysfunction in healthy medical students exposed to academic stress ^143, 144^, and this was similar to other studies ^145, 146^ with the same strain. A probiotic containing seven different bacterial strains was not effective in reducing stress in healthy petrol workers ^147, 148^. Whether and to what extent specific bacterial strains exert convergent and synergistic effects on the (GI) stress response when combined ^149^ are open and unsolved issues. Another is the fact that probiotic consumption may exert differential effects in men and women depending on nutritional habits on the one hand and microbiota composition on the other ^150^, together with sex differences in the stress response, as discussed above. Whether probiotic consumption or nutritional habits are capable of preventing stress vulnerability or increasing stress resilience (or both) is currently unknown but warrants further investigation.

## Closing remarks

**Figure 1.**
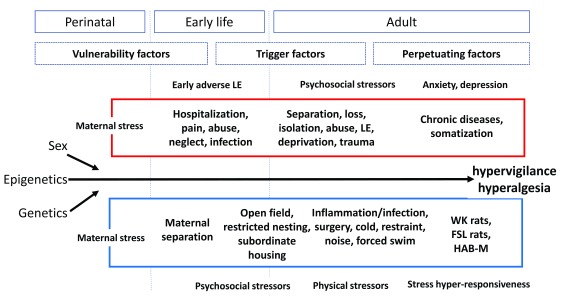
Human (red) and animal (blue) models of stress-induced modulation of visceral sensitivity throughout the life span and for different phases of life (from perinatal to adulthood), together with contributions from genetics/epigenetics and sex FSL, Flinders Sensitive Line; HAB-M, high-anxiety-related-behavior mice; LE, life events; WK, Wistar Kyoto.

Figure 1 is an attempt to summarize current knowledge from animal and human studies and condense it into a scheme of where, when, and how different types of stress may affect central and peripheral functions, mediated by the enteric nervous system or the CNS or both along the gut-brain axis ^151^.

As is evident from the amount of literature published in the last few years, the stress concept (or GAS) has not only survived in gastroenterology, especially the rise of *Helicobacter pylori* as a *conditio sine qua non* mediator of (stress) ulcer formation, but also gained even wider acceptance than in the times of Hans Selye, not the least through his pupils and successors and ongoing research. It is arguably the major concept to explain the cause and course of functional bowel disorders of IBS type (that is, for visceral hypersensitivity and hypervigilance). Translational animal stress models used nowadays simulate much better than ever before the stressors that affect human health in general and GI functions specifically, explain sex differences as they are found in epidemiological data on functional GI disorders, and pave the way for a better understanding of how stress affects the brain in health and disease. As was pointed out recently, “the gastrointestinal system is an ideal model to analyze the interaction between our genes, emotions and the gut microbiota. … an integrated approach … is the next frontier that awaits the gastroenterologist to prevent and treat GI disorders” ^152^.

## References

[1] Selye H: A syndrome produced by diverse nocuous agents. 1936. *J Neuropsychiatry Clin Neurosci.* 1998;10(2):230–1. 10.1176/jnp.10.2.230a 9722327

[2] Selye H: The general adaptation syndrome and the diseases of adaptation. *J Clin Endocrinol Metab.* 1946;6:117–230. 10.1210/jcem-6-2-117 21025115

[3] Guth PH Kaunitz JD: Personal reminiscences about Morton Grossman and the founding of the Center for Ulcer Research and Education (CURE). *Am J Physiol Gastrointest Liver Physiol.* 2008;294(5):G1109–13. 10.1152/ajpgi.00594.2007 18356532

[4] McEwen BS: Protective and damaging effects of stress mediators. *N Engl J Med.* 1998;338(3):171–9. 10.1056/NEJM199801153380307 9428819

[5] Goldstein DS McEwen B: Allostasis, homeostats, and the nature of stress. *Stress.* 2002;5(1):55–8. 10.1080/102538902900012345 12171767

[6] Ursin H Eriksen HR: The cognitive activation theory of stress. *Psychoneuroendocrinology.* 2004;29(5):567–92. 10.1016/S0306-4530(03)00091-X 15041082

[7] de Kloet ER Joëls M Holsboer F: Stress and the brain: from adaptation to disease. *Nat Rev Neurosci.* 2005;6(6):463–75. 10.1038/nrn1683 15891777

[8] Kalisch R Müller MB Tüscher O: A conceptual framework for the neurobiological study of resilience. *Behav Brain Sci.* 2015;38:e92. 10.1017/S0140525X1400082X 25158686

[9] Fukudo S: Stress and visceral pain: focusing on irritable bowel syndrome. *Pain.* 2013;154(Suppl 1):S63–70. 10.1016/j.pain.2013.09.008 24021863

[10] Tanaka Y Kanazawa M Fukudo S: Biopsychosocial model of irritable bowel syndrome. *J Neurogastroenterol Motil.* 2011;17(2):131–9. 10.5056/jnm.2011.17.2.131 21602989 PMC3093004

[11] Van Oudenhove L Crowell MD Drossman DA: Biopsychosocial Aspects of Functional Gastrointestinal Disorders. *Gastroenterology.* 2016;150(6):1355–1367.e2, pii: S0016-5085(16)00218-3. 10.1053/j.gastro.2016.02.027 27144624 PMC8809487

[12] Bonaz BL Bernstein CN: Brain-gut interactions in inflammatory bowel disease. *Gastroenterology.* 2013;144(1):36–49. 10.1053/j.gastro.2012.10.003 23063970

[13] Goodhand JR Wahed M Mawdsley JE: Mood disorders in inflammatory bowel disease: relation to diagnosis, disease activity, perceived stress, and other factors. *Inflamm Bowel Dis.* 2012;18(12):2301–9. 10.1002/ibd.22916 22359369

[14] Bonkovsky HL: On stress and the liver: a chicken and egg conundrum. *Gastroenterology.* 2015;148(5):894–7. 10.1053/j.gastro.2015.03.024 25805419

[15] Dorn SD Hernandez L Minaya MT: Psychosocial factors are more important than disease activity in determining gastrointestinal symptoms and health status in adults at a celiac disease referral center. *Dig Dis Sci.* 2010;55(11):3154–63. 10.1007/s10620-010-1342-y 20668941

[16] Mårild K Frostell AS Ludvigsson JF: Psychological stress and coeliac disease in childhood: a cohort study. *BMC Gastroenterol.* 2010;10:106. 10.1186/1471-230X-10-106 20840747 PMC2945988

[17] Barone FC Deegan JF Price WJ: Cold-restraint stress increases rat fecal pellet output and colonic transit. *Am J Physiol.* 1990;258(3 Pt 1):G329–37. 2316647 10.1152/ajpgi.1990.258.3.G329

[18] Gue M Fioramonti J Bueno L: Comparative influences of acoustic and cold stress on gastrointestinal transit in mice. *Am J Physiol.* 1987;253(2 Pt 1):G124–8. 3497584 10.1152/ajpgi.1987.253.2.G124

[19] Enck P Merlin V Erckenbrecht JF: Stress effects on gastrointestinal transit in the rat. *Gut.* 1989;30(4):455–9. 10.1136/gut.30.4.455 2714679 PMC1434046

[20] Coutinho SV Plotsky PM Sablad M: Neonatal maternal separation alters stress-induced responses to viscerosomatic nociceptive stimuli in rat. *Am J Physiol Gastrointest Liver Physiol.* 2002;282(2):G307–16. 10.1152/ajpgi.00240.2001 11804852

[21] Walker CD Bath KG Joels M: Chronic early life stress induced by limited bedding and nesting (LBN) material in rodents: critical considerations of methodology, outcomes and translational potential. *Stress.* 2017:20(5):421–448. 10.1080/10253890.2017.1343296 28617197 PMC5705407

[22] Goodman WK Janson J Wolf JM: Meta-analytical assessment of the effects of protocol variations on cortisol responses to the Trier Social Stress Test. *Psychoneuroendocrinology.* 2017;80:26–35. 10.1016/j.psyneuen.2017.02.030 28292684

[23] Kirschbaum C Pirke KM Hellhammer DH: The 'Trier Social Stress Test'--a tool for investigating psychobiological stress responses in a laboratory setting. *Neuropsychobiology.* 1993;28(1-2):76–81. 10.1159/000119004 8255414

[24] Elsenbruch S Lucas A Holtmann G: Public speaking stress-induced neuroendocrine responses and circulating immune cell redistribution in irritable bowel syndrome. *Am J Gastroenterol.* 2006;101(10):2300–7. 10.1111/j.1572-0241.2006.00837.x 16952284

[25] Elsenbruch S Rosenberger C Bingel U: Patients with irritable bowel syndrome have altered emotional modulation of neural responses to visceral stimuli. *Gastroenterology.* 2010;139(4):1310–9. 10.1053/j.gastro.2010.06.054 20600024

[26] Kennedy PJ Cryan JF Quigley EM: A sustained hypothalamic-pituitary-adrenal axis response to acute psychosocial stress in irritable bowel syndrome. *Psychol Med.* 2014;44(14):3123–34. 10.1017/S003329171400052X 25065954

[27] Langhorst J Cobelens PM Kavelaars A: Stress-related peripheral neuroendocrine-immune interactions in women with ulcerative colitis. *Psychoneuroendocrinology.* 2007;32(8–10):1086–96. 10.1016/j.psyneuen.2007.09.003 17933470

[28] Roderigo T Benson S Schöls M: Effects of acute psychological stress on placebo and nocebo responses in a clinically relevant model of visceroception. *Pain.* 2017;158(8):1489–98. 10.1097/j.pain.0000000000000940 28471874

[29] Rosenberger C Elsenbruch S Scholle A: Effects of psychological stress on the cerebral processing of visceral stimuli in healthy women. *Neurogastroenterol Motil.* 2009;21(7):740–e45. 10.1111/j.1365-2982.2009.01295.x 19368654

[30] Vanuytsel T van Wanrooy S Vanheel H: Psychological stress and corticotropin-releasing hormone increase intestinal permeability in humans by a mast cell-dependent mechanism. *Gut.* 2014;63(8):1293–9. 10.1136/gutjnl-2013-305690 24153250

[31] Alonso C Guilarte M Vicario M: Acute experimental stress evokes a differential gender-determined increase in human intestinal macromolecular permeability. *Neurogastroenterol Motil.* 2012;24(8):740–6,e348–9. 10.1111/j.1365-2982.2012.01928.x 22625665

[32] Dickhaus B Mayer EA Firooz N: Irritable bowel syndrome patients show enhanced modulation of visceral perception by auditory stress. *Am J Gastroenterol.* 2003;98(1):135–43. 10.1111/j.1572-0241.2003.07156.x 12526949

[33] Murray CD Flynn J Ratcliffe L: Effect of acute physical and psychological stress on gut autonomic innervation in irritable bowel syndrome. *Gastroenterology.* 2004;127(6):1695–703. 10.1053/j.gastro.2004.08.057 15578507

[34] Posserud I Agerforz P Ekman R: Altered visceral perceptual and neuroendocrine response in patients with irritable bowel syndrome during mental stress. *Gut.* 2004;53(8):1102–8. 10.1136/gut.2003.017962 15247175 PMC1774150

[35] Broers C Melchior C Van Oudenhove L: The effect of intravenous corticotropin-releasing hormone administration on esophageal sensitivity and motility in health. *Am J Physiol Gastrointest Liver Physiol.* 2017;312(5):G526–G534. 10.1152/ajpgi.00437.2016 28336550

[36] Hubbard CS Labus JS Bueller J: Corticotropin-releasing factor receptor 1 antagonist alters regional activation and effective connectivity in an emotional-arousal circuit during expectation of abdominal pain. *J Neurosci.* 2011;31(35):12491–500. 10.1523/JNEUROSCI.1860-11.2011 21880911 PMC3399687

[37] Labus JS Hubbard CS Bueller J: Impaired emotional learning and involvement of the corticotropin-releasing factor signaling system in patients with irritable bowel syndrome. *Gastroenterology.* 2013;145(6):1253–61.e1–3. 10.1053/j.gastro.2013.08.016 23954313 PMC4069031

[38] Tanaka Y Kanazawa M Kano M: Differential Activation in Amygdala and Plasma Noradrenaline during Colorectal Distention by Administration of Corticotropin-Releasing Hormone between Healthy Individuals and Patients with Irritable Bowel Syndrome. *PLoS One.* 2016;11(7):e0157347. 10.1371/journal.pone.0157347 27448273 PMC4957789

[39] Schneiderman N Ironson G Siegel SD: Stress and health: psychological, behavioral, and biological determinants. *Annu Rev Clin Psychol.* 2005;1:607–28. 10.1146/annurev.clinpsy.1.102803.144141 17716101 PMC2568977

[40] Brzozowski B Mazur-Bialy A Pajdo R: Mechanisms by which Stress Affects the Experimental and Clinical Inflammatory Bowel Disease (IBD): Role of Brain-Gut Axis. *Curr Neuropharmacol.* 2016;14(8):892–900. 10.2174/1570159X14666160404124127 27040468 PMC5333596

[41] Spiegel BM Khanna D Bolus R: Understanding gastrointestinal distress: a framework for clinical practice. *Am J Gastroenterol.* 2011;106(3):380–5. 10.1038/ajg.2010.383 21378758 PMC4275100

[42] Schulz P Schlotz W: Trierer Inventar zur Erfassung von chronischem Streß (TICS): Skalenkonstruktion, teststatistische Überprüfung und Validierung der Skala Arbeitsüberlastung. *Diagnostica.* 1999;45:8–19. 10.1026//0012-1924.45.1.8

[43] Levenstein S Prantera C Varvo V: Development of the Perceived Stress Questionnaire: a new tool for psychosomatic research. *J Psychosom Res.* 1993;37(1):19–32. 10.1016/0022-3999(93)90120-5 8421257

[44] Keszthelyi D Troost FJ Masclee AA: Irritable bowel syndrome: methods, mechanisms, and pathophysiology. Methods to assess visceral hypersensitivity in irritable bowel syndrome. *Am J Physiol Gastrointest Liver Physiol.* 2012;303(2):G141–54. 10.1152/ajpgi.00060.2012 22595988

[45] Chaloner A Greenwood-Van Meerveld B: Early life adversity as a risk factor for visceral pain in later life: importance of sex differences. *Front Neurosci.* 2013;7:13. 10.3389/fnins.2013.00013 23407595 PMC3570767

[46] Stam R Ekkelenkamp K Frankhuijzen AC: Long-lasting changes in central nervous system responsivity to colonic distention after stress in rats. *Gastroenterology.* 2002;123(4):1216–25. 10.1053/gast.2002.36029 12360483

[47] Hyland NP O'Mahony SM O'Malley D: Early-life stress selectively affects gastrointestinal but not behavioral responses in a genetic model of brain-gut axis dysfunction. *Neurogastroenterol Motil.* 2015;27(1):105–13. 10.1111/nmo.12486 25443141

[48] Pohl CS Medland JE Mackey E: Early weaning stress induces chronic functional diarrhea, intestinal barrier defects, and increased mast cell activity in a porcine model of early life adversity. *Neurogastroenterol Motil.* 2017;29(11). 10.1111/nmo.13118 28573751 PMC5650513

[49] Medland JE Pohl CS Edwards LL: Early life adversity in piglets induces long-term upregulation of the enteric cholinergic nervous system and heightened, sex-specific secretomotor neuron responses. *Neurogastroenterol Motil.* 2016;28(9):1317–29. 10.1111/nmo.12828 27134125 PMC5002263

[50] Aguggia JP Suárez MM Rivarola MA: Early maternal separation: neurobehavioral consequences in mother rats. *Behav Brain Res.* 2013;248:25–31. 10.1016/j.bbr.2013.03.040 23567892

[51] Silveira PP Benetti Cda S Portella AK: Brief daily postpartum separations from the litter alter dam response to psychostimulants and to stress. *Braz J Med Biol Res.* 2013;46(5):426–32. 10.1590/1414-431X20132784 23739746 PMC3854400

[52] Winston JH Li Q Sarna SK: Chronic prenatal stress epigenetically modifies spinal cord BDNF expression to induce sex-specific visceral hypersensitivity in offspring. *Neurogastroenterol Motil.* 2014;26(5):715–30. 10.1111/nmo.12326 24588943 PMC3997587

[53] van den Wijngaard RM Stanisor OI van Diest SA: Susceptibility to stress induced visceral hypersensitivity in maternally separated rats is transferred across generations. *Neurogastroenterol Motil.* 2013;25(12):e780–90. 10.1111/nmo.12202 23965154

[54] Dinan TG Cryan J Shanahan F: IBS: An epigenetic perspective. *Nat Rev Gastroenterol Hepatol.* 2010;7(8):465–71. 10.1038/nrgastro.2010.99 20585338

[55] Macrì S Zoratto F Laviola G: Early-stress regulates resilience, vulnerability and experimental validity in laboratory rodents through mother-offspring hormonal transfer. *Neurosci Biobehav Rev.* 2011;35(7):1534–43. 10.1016/j.neubiorev.2010.12.014 21216260

[56] Moussaoui N Jacobs JP Larauche M: Chronic Early-life Stress in Rat Pups Alters Basal Corticosterone, Intestinal Permeability, and Fecal Microbiota at Weaning: Influence of Sex. *J Neurogastroenterol Motil.* 2017;23(1):135–43. 10.5056/jnm16105 27829577 PMC5216644

[57] West C Wu RY Wong A: *Lactobacillus rhamnosus* strain JB-1 reverses restraint stress-induced gut dysmotility. *Neurogastroenterol Motil.* 2017;29(1). 10.1111/nmo.12903 27381257

[58] Holzer P Reichmann F Farzi A: Neuropeptide Y, peptide YY and pancreatic polypeptide in the gut-brain axis. *Neuropeptides.* 2012;46(6):261–74. 10.1016/j.npep.2012.08.005 22979996 PMC3516703

[59] Overstreet DH Wegener G: The flinders sensitive line rat model of depression--25 years and still producing. *Pharmacol Rev.* 2013;65(1):143–55. 10.1124/pr.111.005397 23319547

[60] Krömer SA Kessler MS Milfay D: Identification of glyoxalase-I as a protein marker in a mouse model of extremes in trait anxiety. *J Neurosci.* 2005;25(17):4375–84. 10.1523/JNEUROSCI.0115-05.2005 15858064 PMC6725100

[61] Bassett S Young W Fraser K: Stress differentially alters the plasma and brain metabolomes and caecal microbiome in Wistar Kyoto and Sprague Dawley rats. *Neurogastroenterol Motil.* 2017;29(Suppl 2):60 Reference Source

[62] Savignac HM Dinan TG Cryan JF: Resistance to early-life stress in mice: effects of genetic background and stress duration. *Front Behav Neurosci.* 2011;5:13. 10.3389/fnbeh.2011.00013 21519375 PMC3075880

[63] Zannas AS West AE: Epigenetics and the regulation of stress vulnerability and resilience. *Neuroscience.* 2014;264:157–70. 10.1016/j.neuroscience.2013.12.003 24333971 PMC3959582

[64] Singh-Taylor A Korosi A Molet J: Synaptic rewiring of stress-sensitive neurons by early-life experience: a mechanism for resilience? *Neurobiol Stress.* 2015;1:109–15. 10.1016/j.ynstr.2014.10.007 25530985 PMC4267062

[65] Daskalakis NP Bagot RC Parker KJ: The three-hit concept of vulnerability and resilience: toward understanding adaptation to early-life adversity outcome. *Psychoneuroendocrinology.* 2013;38(9):1858–73. 10.1016/j.psyneuen.2013.06.008 23838101 PMC3773020

[66] Park SH Naliboff BD Shih W: Resilience is decreased in irritable bowel syndrome and associated with symptoms and cortisol response. *Neurogastroenterol Motil.* 2017. 10.1111/nmo.13155 28718999 PMC5739983

[67] Gandhi S Jedel S Hood MM: The relationship between coping, health competence and patient participation among patients with inactive inflammatory bowel disease. *J Crohns Colitis.* 2014;8(5):401–8. 10.1016/j.crohns.2013.10.005 24230968

[68] Petrak F Hardt J Clement T: Impaired health-related quality of life in inflammatory bowel diseases: psychosocial impact and coping styles in a national German sample. *Scand J Gastroenterol.* 2001;36(4):375–82. 10.1080/00365520118423 11336162

[69] van Tilburg MA Claar RL Romano JM: Role of Coping With Symptoms in Depression and Disability: Comparison Between Inflammatory Bowel Disease and Abdominal Pain. *J Pediatr Gastroenterol Nutr.* 2015;61(4):431–6. 10.1097/MPG.0000000000000841 25944213 PMC4581886

[70] Houghton LA Heitkemper M Crowell M: Age, Gender and Women's Health and the Patient. *Gastroenterology.* 2016;150(6):1332–1343.e4, pii: S0016-5085(16)00183-9. 10.1053/j.gastro.2016.02.017 27144622

[71] Million M Larauche M: Stress, sex, and the enteric nervous system. *Neurogastroenterol Motil.* 2016;28(9):1283–9. 10.1111/nmo.12937 27561694 PMC5003424

[72] Prusator DK Greenwood-Van Meerveld B: Gender specific effects of neonatal limited nesting on viscerosomatic sensitivity and anxiety-like behavior in adult rats. *Neurogastroenterol Motil.* 2015;27(1):72–81. 10.1111/nmo.12472 25394875

[73] Bale TL Epperson CN: Sex differences and stress across the lifespan. *Nat Neurosci.* 2015;18(10):1413–20. 10.1038/nn.4112 26404716 PMC4620712

[74] Mueller BR Bale TL: Sex-specific programming of offspring emotionality after stress early in pregnancy. *J Neurosci.* 2008;28(36):9055–65. 10.1523/JNEUROSCI.1424-08.2008 18768700 PMC2731562

[75] Walker MM Keely SJ Scott RJ: Genetics, Mucosal Inflammation, and the Environment in Post-Infectious Chronic Gut Syndromes. *Am J Gastroenterol Suppl.* 2016;3:46–51. 10.1038/ajgsup.2016.14

[76] Moussaoui N Larauche M Biraud M: Limited Nesting Stress Alters Maternal Behavior and *In Vivo* Intestinal Permeability in Male Wistar Pup Rats. *PLoS One.* 2016;11(5):e0155037. 10.1371/journal.pone.0155037 27149676 PMC4858303

[77] Larauche M Mulak A Kim YS: Visceral analgesia induced by acute and repeated water avoidance stress in rats: sex difference in opioid involvement. *Neurogastroenterol Motil.* 2012;24(11):1031–e547. 10.1111/j.1365-2982.2012.01980.x 22776034 PMC3470786

[78] Mackey E Ayyadurai S Pohl CS: Sexual dimorphism in the mast cell transcriptome and the pathophysiological responses to immunological and psychological stress. *Biol Sex Differ.* 2016;7:60. 10.1186/s13293-016-0113-7 27895892 PMC5120457

[79] Boeckxstaens G Camilleri M Sifrim D: Fundamentals of Neurogastroenterology: Physiology/Motility - Sensation. *Gastroenterology.* 2016;150(6):1292–1304.e2. pii: S0016-5085(16)00221-3. 10.1053/j.gastro.2016.02.030 27144619

[80] Van Oudenhove L Aziz Q: The role of psychosocial factors and psychiatric disorders in functional dyspepsia. *Nat Rev Gastroenterol Hepatol.* 2013;10(3):158–67. 10.1038/nrgastro.2013.10 23358396

[81] Targownik LE Sexton KA Bernstein MT: The Relationship Among Perceived Stress, Symptoms, and Inflammation in Persons With Inflammatory Bowel Disease. *Am J Gastroenterol.* 2015;110(7):1001–12. quiz 1013. 10.1038/ajg.2015.147 26077178

[82] Koloski NA Jones M Talley NJ: Evidence that independent gut-to-brain and brain-to-gut pathways operate in the irritable bowel syndrome and functional dyspepsia: a 1-year population-based prospective study. *Aliment Pharmacol Ther.* 2016;44(6):592–600. 10.1111/apt.13738 27444264

[83] Ly HG Weltens N Tack J: Acute Anxiety and Anxiety Disorders Are Associated With Impaired Gastric Accommodation in Patients With Functional Dyspepsia. *Clin Gastroenterol Hepatol.* 2015;13(9):1584–91.e3. 10.1016/j.cgh.2015.03.032 25869636

[84] Van Oudenhove L Vandenberghe J Geeraerts B: Relationship between anxiety and gastric sensorimotor function in functional dyspepsia. *Psychosom Med.* 2007;69(5):455–63. 10.1097/PSY.0b013e3180600a4a 17556644

[85] Hveem K Hausken T Svebak S: Gastric antral motility in functional dyspepsia. Effect of mental stress and cisapride. *Scand J Gastroenterol.* 1996;31(5):452–7. 10.3109/00365529609006764 8734341

[86] Vanner S Greenwood-Van Meerveld B Mawe G: Fundamentals of Neurogastroenterology: Basic Science. *Gastroenterology.* 2016;150(6):1280–1291, pii: S0016-5085(16)00184-0. 10.1053/j.gastro.2016.02.018 27144618 PMC5673591

[87] Al Omran Y Aziz Q: Functional brain imaging in gastroenterology: to new beginnings. *Nat Rev Gastroenterol Hepatol.* 2014;11(9):565–76. 10.1038/nrgastro.2014.89 24912384

[88] Lee IS Wang H Chae Y: Functional neuroimaging studies in functional dyspepsia patients: a systematic review. *Neurogastroenterol Motil.* 2016;28(6):793–805. 10.1111/nmo.12793 26940430

[89] Mayer EA Gupta A Kilpatrick LA: Imaging brain mechanisms in chronic visceral pain. *Pain.* 2015;156(Suppl 1):S50–63. 10.1097/j.pain.0000000000000106 25789437 PMC4428597

[90] Coen SJ Yágüez L Aziz Q: Negative mood affects brain processing of visceral sensation. *Gastroenterology.* 2009;137(1):253–61,261.e1–2. 10.1053/j.gastro.2009.02.052 19582887

[91] Elsenbruch S Rosenberger C Enck P: Affective disturbances modulate the neural processing of visceral pain stimuli in irritable bowel syndrome: an fMRI study. *Gut.* 2010;59(4):489–95. 10.1136/gut.2008.175000 19651629

[92] Van Oudenhove L Vandenberghe J Dupont P: Abnormal regional brain activity during rest and (anticipated) gastric distension in functional dyspepsia and the role of anxiety: a H _2_ ^15^O-PET study. *Am J Gastroenterol.* 2010;105(4):913–24. 10.1038/ajg.2010.39 20160711

[93] Icenhour A Langhorst J Benson S: Neural circuitry of abdominal pain-related fear learning and reinstatement in irritable bowel syndrome. *Neurogastroenterol Motil.* 2015;27(1):114–27. 10.1111/nmo.12489 25557224

[94] Icenhour A Labrenz F Ritter C: Learning by experience? Visceral pain-related neural and behavioral responses in a classical conditioning paradigm. *Neurogastroenterol Motil.* 2017;29(6):e13026. 10.1111/nmo.13026 28177183

[95] Agostini A Filippini N Cevolani D: Brain functional changes in patients with ulcerative colitis: a functional magnetic resonance imaging study on emotional processing. *Inflamm Bowel Dis.* 2011;17(8):1769–77. 10.1002/ibd.21549 21744432

[96] Rubio A Pellissier S Van Oudenhove L: Brain responses to uncertainty about upcoming rectal discomfort in quiescent Crohn's disease - a fMRI study. *Neurogastroenterol Motil.* 2016;28(9):1419–32. 10.1111/nmo.12844 27132547

[97] Agostini A Filippini N Benuzzi F: Functional magnetic resonance imaging study reveals differences in the habituation to psychological stress in patients with Crohn's disease versus healthy controls. *J Behav Med.* 2013;36(5):477–87. 10.1007/s10865-012-9441-1 22752251

[98] Agostini A Ballotta D Righi S: Stress and brain functional changes in patients with Crohn's disease: A functional magnetic resonance imaging study. *Neurogastroenterol Motil.* 2017;29(10):1–10. 10.1111/nmo.13108 28560758

[99] Baliki MN Apkarian AV: Nociception, Pain, Negative Moods, and Behavior Selection. *Neuron.* 2015;87(3):474–91. 10.1016/j.neuron.2015.06.005 26247858 PMC4529956

[100] Dejean C Courtin J Rozeske RR: Neuronal Circuits for Fear Expression and Recovery: Recent Advances and Potential Therapeutic Strategies. *Biol Psychiatry.* 2015;78(5):298–306. 10.1016/j.biopsych.2015.03.017 25908496

[101] Craske MG Wolitzky-Taylor KB Labus J: A cognitive-behavioral treatment for irritable bowel syndrome using interoceptive exposure to visceral sensations. *Behav Res Ther.* 2011;49(6–7):413–21. 10.1016/j.brat.2011.04.001 21565328 PMC3100429

[102] Ljótsson B Hesser H Andersson E: Provoking symptoms to relieve symptoms: a randomized controlled dismantling study of exposure therapy in irritable bowel syndrome. *Behav Res Ther.* 2014;55:27–39. 10.1016/j.brat.2014.01.007 24584055

[103] Vlaeyen JW de Jong J Geilen M: The treatment of fear of movement/(re)injury in chronic low back pain: further evidence on the effectiveness of exposure *in vivo*. *Clin J Pain.* 2002;18(4):251–61. 10.1097/00002508-200207000-00006 12131067

[104] Williams AE Rhudy JL: The influence of conditioned fear on human pain thresholds: does preparedness play a role? *J Pain.* 2007;8(7):598–606. 10.1016/j.jpain.2007.03.004 17524956

[105] Jensen K Kirsch I Odmalm S: Classical conditioning of analgesic and hyperalgesic pain responses without conscious awareness. *Proc Natl Acad Sci U S A.* 2015;112(25):7863–7. 10.1073/pnas.1504567112 25979940 PMC4485119

[106] Overmier JB: Sensitization, conditioning, and learning: can they help us understand somatization and disability? *Scand J Psychol.* 2002;43(2):105–12. 10.1111/1467-9450.00275 12004947

[107] Zaman J Vlaeyen JW Van Oudenhove L: Associative fear learning and perceptual discrimination: a perceptual pathway in the development of chronic pain. *Neurosci Biobehav Rev.* 2015;51:118–25. 10.1016/j.neubiorev.2015.01.009 25603316

[108] Meulders A Jans A Vlaeyen JW: Differences in pain-related fear acquisition and generalization: an experimental study comparing patients with fibromyalgia and healthy controls. *Pain.* 2015;156(1):108–22. 10.1016/j.pain.0000000000000016 25599307

[109] Lowén MB Mayer E Tillisch K: Deficient habituation to repeated rectal distensions in irritable bowel syndrome patients with visceral hypersensitivity. *Neurogastroenterol Motil.* 2015;27(5):646–55. 10.1111/nmo.12537 25777251

[110] Icenhour A Kattoor J Benson S: Neural circuitry underlying effects of context on human pain-related fear extinction in a renewal paradigm. *Hum Brain Mapp.* 2015;36(8):3179–93. 10.1002/hbm.22837 26058893 PMC6869065

[111] Elsenbruch S Wolf OT: Could Stress Contribute to Pain-Related Fear in Chronic Pain? *Front Behav Neurosci.* 2015;9:340. 10.3389/fnbeh.2015.00340 26733831 PMC4681808

[112] Nekovarova T Yamamotova A Vales K: Common mechanisms of pain and depression: are antidepressants also analgesics? *Front Behav Neurosci.* 2014;8:99. 10.3389/fnbeh.2014.00099 24723864 PMC3971163

[113] Li S Wang Z Yang Y: *Lachnospiraceae* shift in the microbial community of mice faecal sample effects on water immersion restraint stress. *AMB Express.* 2017;7(1):82. 10.1186/s13568-017-0383-4 28417435 PMC5393979

[114] Jašarević E Howard CD Misic AM: Stress during pregnancy alters temporal and spatial dynamics of the maternal and offspring microbiome in a sex-specific manner. *Sci Rep.* 2017;7: 44182. 10.1038/srep44182 28266645 PMC5339804

[115] Jašarević E Morrison KE Bale TL: Sex differences in the gut microbiome-brain axis across the lifespan. *Philos Trans R Soc Lond B Biol Sci.* 2016;371(1688):20150122. 10.1098/rstb.2015.0122 26833840 PMC4785905

[116] Hemmings SMJ Malan-Müller S van den Heuvel LL: The Microbiome in Posttraumatic Stress Disorder and Trauma-Exposed Controls: An Exploratory Study. *Psychosom Med.* 2017;79(8):936–46. 10.1097/PSY.0000000000000512 28700459 PMC5763914

[117] Rajilić-Stojanović M Jonkers DM Salonen A: Intestinal microbiota and diet in IBS: causes, consequences, or epiphenomena? *Am J Gastroenterol.* 2015;110(2):278–87. 10.1038/ajg.2014.427 25623659 PMC4317767

[118] Jiang H Ling Z Zhang Y: Altered fecal microbiota composition in patients with major depressive disorder. *Brain Behav Immun.* 2015;48:186–94. 10.1016/j.bbi.2015.03.016 25882912

[119] Mack I Cuntz U Grämer C: Weight gain in anorexia nervosa does not ameliorate the faecal microbiota, branched chain fatty acid profiles, and gastrointestinal complaints. *Sci Rep.* 2016;6: 26752. 10.1038/srep26752 27229737 PMC4882621

[120] Sharon G Sampson TR Geschwind DH: The Central Nervous System and the Gut Microbiome. *Cell.* 2016;167(4):915–32. 10.1016/j.cell.2016.10.027 27814521 PMC5127403

[121] Clark A Mach N: The Crosstalk between the Gut Microbiota and Mitochondria during Exercise. *Front Physiol.* 2017;8:319. 10.3389/fphys.2017.00319 28579962 PMC5437217

[122] Karl JP Margolis LM Madslien EH: Changes in intestinal microbiota composition and metabolism coincide with increased intestinal permeability in young adults under prolonged physiological stress. *Am J Physiol Gastrointest Liver Physiol.* 2017;312(6):G559–G571. 10.1152/ajpgi.00066.2017 28336545

[123] Barton W Penney NC Cronin O: The microbiome of professional athletes differs from that of more sedentary subjects in composition and particularly at the functional metabolic level. *Gut.* 2017; pii: gutjnl-2016-313627. 10.1136/gutjnl-2016-313627 28360096

[124] Bressa C Bailén-Andrino M Pérez-Santiago J: Differences in gut microbiota profile between women with active lifestyle and sedentary women. *PLoS One.* 2017;12(2):e0171352. 10.1371/journal.pone.0171352 28187199 PMC5302835

[125] Paulsen JA Ptacek TS Carter SJ: Gut microbiota composition associated with alterations in cardiorespiratory fitness and psychosocial outcomes among breast cancer survivors. *Support Care Cancer.* 2017;25(5):1563–70. 10.1007/s00520-016-3568-5 28064384 PMC5380600

[126] Wang H Lee IS Braun C: Effect of Probiotics on Central Nervous System Functions in Animals and Humans: A Systematic Review. *J Neurogastroenterol Motil.* 2016;22(4):589–605. 10.5056/jnm16018 27413138 PMC5056568

[127] Bravo JA Forsythe P Chew MV: Ingestion of *Lactobacillus* strain regulates emotional behavior and central GABA receptor expression in a mouse via the vagus nerve. *Proc Natl Acad Sci U S A.* 2011;108(38):16050–5. 10.1073/pnas.1102999108 21876150 PMC3179073

[128] Kelly JR Allen AP Temko A: Lost in translation? The potential psychobiotic *Lactobacillus rhamnosus* (JB-1) fails to modulate stress or cognitive performance in healthy male subjects. *Brain Behav Immun.* 2017;61:50–9. 10.1016/j.bbi.2016.11.018 27865949

[129] Nonaka Y Izumo T Maekawa T: Anti-stress effect of the *Lactobacillus pentosus* strain S-PT84 in mice. *Biosci Microbiota Food Health.* 2017;36(3):121–8. 10.12938/bmfh.17-003 28748133 PMC5510157

[130] Barrera-Bugueño C Realini O Escobar-Luna J: Anxiogenic effects of a Lactobacillus, inulin and the synbiotic on healthy juvenile rats. *Neuroscience.* 2017;359:18–29. 10.1016/j.neuroscience.2017.06.064 28694176

[131] Liang S Wang T Hu X: Administration of *Lactobacillus helveticus* NS8 improves behavioral, cognitive, and biochemical aberrations caused by chronic restraint stress. *Neuroscience.* 2015;310:561–77. 10.1016/j.neuroscience.2015.09.033 26408987

[132] Pusceddu MM El Aidy S Crispie F: N-3 Polyunsaturated Fatty Acids (PUFAs) Reverse the Impact of Early-Life Stress on the Gut Microbiota. *PLoS One.* 2015;10(10):e0139721. 10.1371/journal.pone.0139721 26426902 PMC4591340

[133] Marin IA Goertz JE Ren T: Microbiota alteration is associated with the development of stress-induced despair behavior. *Sci Rep.* 2017;7:43859. 10.1038/srep43859 28266612 PMC5339726

[134] Yang C Fujita Y Ren Q: *Bifidobacterium* in the gut microbiota confer resilience to chronic social defeat stress in mice. *Sci Rep.* 2017;7:45942. 10.1038/srep45942 28368029 PMC5377462

[135] Larauche M Mulak A Yuan P: Stress-induced visceral analgesia assessed non-invasively in rats is enhanced by prebiotic diet. *World J Gastroenterol.* 2012;18(3):225–36. 10.3748/wjg.v18.i3.225 22294825 PMC3261539

[136] Burokas A Arboleya S Moloney RD: Targeting the Microbiota-Gut-Brain Axis: Prebiotics Have Anxiolytic and Antidepressant-like Effects and Reverse the Impact of Chronic Stress in Mice. *Biol Psychiatry.* 2017;82(7):472–87. 10.1016/j.biopsych.2016.12.031 28242013

[137] Wang HY Enck P Braun C: Effects of rifaximin on neural responses to social stress: A pilot experiment. *Neurogastroenterol Motil.* 2017;29(Suppl 2):83–84.

[138] Wang H Braun C Enck P: How the brain reacts to social stress (exclusion) - A scoping review. *Neurosci Biobehav Rev.* 2017;80:80–8. 10.1016/j.neubiorev.2017.05.012 28535967

[139] Savignac HM Kiely B Dinan TG: *Bifidobacteria* exert strain-specific effects on stress-related behavior and physiology in BALB/c mice. *Neurogastroenterol Motil.* 2014;26(11):1615–27. 10.1111/nmo.12427 25251188

[140] Savignac HM Tramullas M Kiely B: Bifidobacteria modulate cognitive processes in an anxious mouse strain. *Behav Brain Res.* 2015;287:59–72. 10.1016/j.bbr.2015.02.044 25794930

[141] Allen AP Hutch W Borre YE: *Bifidobacterium longum* 1714 as a translational psychobiotic: modulation of stress, electrophysiology and neurocognition in healthy volunteers. *Transl Psychiatry.* 2016;6(11):e939. 10.1038/tp.2016.191 27801892 PMC5314114

[142] Allen AP Clarke G Cryan JF: *Bifidobacterium infantis 35624* and other probiotics in the management of irritable bowel syndrome. Strain specificity, symptoms, and mechanisms. *Curr Med Res Opin.* 2017;33(7):1349–51. 10.1080/03007995.2017.1322571 28436237

[143] Kato-Kataoka A Nishida K Takada M: Fermented Milk Containing *Lactobacillus casei* Strain Shirota Preserves the Diversity of the Gut Microbiota and Relieves Abdominal Dysfunction in Healthy Medical Students Exposed to Academic Stress. *Appl Environ Microbiol.* 2016;82(12):3649–58. 10.1128/AEM.04134-15 27208120 PMC4959178

[144] Kato-Kataoka A Nishida K Takada M: Fermented milk containing *Lactobacillus casei* strain Shirota prevents the onset of physical symptoms in medical students under academic examination stress. *Benef Microbes.* 2016;7(2):153–6. 10.3920/BM2015.0100 26689231

[145] Takada M Nishida K Gondo Y: Beneficial effects of *Lactobacillus casei* strain Shirota on academic stress-induced sleep disturbance in healthy adults: a double-blind, randomised, placebo-controlled trial. *Benef Microbes.* 2017;8(2):153–62. 10.3920/BM2016.0150 28443383

[146] Takada M Nishida K Kataoka-Kato A: Probiotic *Lactobacillus casei* strain Shirota relieves stress-associated symptoms by modulating the gut-brain interaction in human and animal models. *Neurogastroenterol Motil.* 2016;28(7):1027–36. 10.1111/nmo.12804 26896291

[147] Mohammadi AA Jazayeri S Khosravi-Darani K: Effects of Probiotics on Biomarkers of Oxidative Stress and Inflammatory Factors in Petrochemical Workers: A Randomized, Double-blind, Placebo-controlled Trial. *Int J Prev Med.* 2015;6(1):82. 10.4103/2008-7802.164146 26445629 PMC4587074

[148] Mohammadi AA Jazayeri S Khosravi-Darani K: The effects of probiotics on mental health and hypothalamic-pituitary-adrenal axis: A randomized, double-blind, placebo-controlled trial in petrochemical workers. *Nutr Neurosci.* 2016;19(9):387–95. 10.1179/1476830515Y.0000000023 25879690

[149] Lomasney KW Cryan JF Hyland NP: Converging effects of a *Bifidobacterium* and *Lactobacillus* probiotic strain on mouse intestinal physiology. *Am J Physiol Gastrointest Liver Physiol.* 2014;307(2):G241–7. 10.1152/ajpgi.00401.2013 24852567

[150] Suzuki Y Ikeda K Sakuma K: Association between Yogurt Consumption and Intestinal Microbiota in Healthy Young Adults Differs by Host Gender. *Front Microbiol.* 2017;8:847. 10.3389/fmicb.2017.00847 28553274 PMC5425481

[151] Larauche M Mulak A Taché Y: Stress-related alterations of visceral sensation: animal models for irritable bowel syndrome study. *J Neurogastroenterol Motil.* 2011;17(3):213–34. 10.5056/jnm.2011.17.3.213 21860814 PMC3155058

[152] Panduro A Rivera-Iñiguez I Sepulveda-Villegas M: Genes, emotions and gut microbiota: The next frontier for the gastroenterologist. *World J Gastroenterol.* 2017;23(17):3030–42. 10.3748/wjg.v23.i17.3030 28533660 PMC5423040

